# Clinical Remarks on Diabetes, and on Delirium Tremens

**Published:** 1872-02

**Authors:** Thos. F. Rochester

**Affiliations:** The Hospital of the Sisters of Charity


### ART. IV.—*Clinical Remarks on Diabetes, and on Delirium Tremens.* By Prof. THOS. F. ROCHESTER, M.D., in the Hospital of the Sisters of Charity.

Reported by EDWARD N. BRUSH, member of the Class.

GENTLEMEN:—

The patient to whom I called your attention in the ward, as suffering from Diabetes, is a young man, nineteen years of age. He has had the disease for a year at least and perhaps longer. During that time he has occasionally passed seven and eight quarts of urine per diem. He is now passing four and five quarts. He was admitted to the Hospital on the 8th of Oct., and since that time has kept constantly to his bed. He is much emaciated, having lost thirty or more pounds.

Diabetes is a disorder in which, with an unusual amount of urinary secretion there is also found in the urine a variable quantity of sugar. This is grape sugar, existing as I have said in variable quantity, some patients will produce one pound, others a pound and a half, and others even two pounds per diem. Besides being in the urine, sugar exists to a greater or less extent in the other secretions and excretions of those having diabetes, as in the saliva, fæces, etc., although this fact has been denied by some physiologists. Diabetes is attended by general debility, great thirst, and red tongue, as you have observed in the young man alluded to. The pulse is variable and unusually slow. In the case of the young man it is but 60 per minute, which is very slow for a youth of nineteen. In Diabetes the skin is dry and almost always cold.

There has been a great amount of ingenuity put forth to ascertain the cause of this disease. When first noticed it was supposed to be a disease of the kidneys. This, it is unnecessary for me to tell you, is not so. It is not a renal disease. After existing for a year or more, diabetes may cause what is termed degeneration of the kidneys, but it, of itself is not a renal disorder.

In an analysis of blood of diabetic patients, sugar is not found permiating the arterial blood. It is not found in the Portal vein, but is found in the Hepathic. It is found in ascending vena cava but not in descending vena cava. It was discovered that on irritating that portion of the brain under the fourth ventricle, sugar was immediately produced in the urine. On ligating the pneumogastric nerve, sugar was no longer found in the urine, but on irritating the brain under the fourth ventricle it was again produced.

It must not be supposed that because sugar exists in the urine that the patient has diabetes, but when the sugar is present permanently and in considerable quantities, it is then indicative of diabetes. The urine of diabetes has great specific gravity, that in this case being 1032, water being 1000. The smell of the urine is like that of new mown hay, and in many cases the odor arising from

Diabetic patients has a sacharine and sickening character. The tests for sugar in the urine which I propose to show you to-day are two in number. The first is called Trommer's test; after adding a few drops of solution of sulphate of copper, pour into the urine in the test tube about half its bulk of liquor potassa. On heating the urine passes from blue to a beautiful green color, and thence to a yellow and dark brown color. The oxide of copper is precipitated. The second test consists in boiling the urine with an equal amount of liquor potassa. The mixture becomes as dark as molasses.

Diabetes proves more rapidly fatal in younger patients. If they do not die from some other disease against which they are not able to cope, they sink from exhaustion produced by the drain on the system. Pulmonary Tuberculosis is very apt to ensue in Diabetic patients.

Knowing that an abundant amount of sugar is being eliminated from the system, the treatment would consist largely in excluding those articles of diet containing sugar and starch. Nearly all kinds of vegetables and fruits are to be withheld, and articles of food consisting wholly or in part of wheat flour, if partaken of at all are to be used with discretion.

Eggs, meat, milk, articles the opposite of those just mentioned are to be used. Some cases have been reported as cured in this manner, but the number is few, the majority being relieved but for a time. Creasote has been recommended. This patient has been taking one drop three times daily.

Strychina has been used with great benefit in connection with simple and proper diet. This patient has been placed on milk diet which has been used in the past few years with marked benefit. An exclusive diet of this kind becomes tiresome and disagreeable, and care should be taken to vary the articles of food prepared from it as much as possible.

In connection with this, the patient is taking the following:

℞ Strychnia, gr. i.

Acid Aceteci, q. s.

Water, ℥ iiss.

M One teaspoonful ter in die.

---

*Delirium Tremens.*—You will doubtless, Gentlemen, remember the patient whom you saw a few days since suffering from an attack of Delirium Tremens. When you saw him he was jocose and seemed in high spirits, but in the course of the afternoon he became wild and delirious, and was with difficulty managed, imagining himself tormented by all sorts of objects both natural and supernatural.

There is not, comparatively, as much Delirium Tremens as formerly, owing no doubt to a diminished consumption of alcohol. The beverage more universally employed by laboring men being beer, containing a less quantity of alcohol. This, if any stimulant at all is needed, which undoubtedly is not the case, is a benefit to the consumers of this beverage.

In the consideration of Delirium Tremens you have to distinguish between two varieties. The first is the Tremulous or Delirium Tremens proper. The second, or Mania a potu, is that variety which is brought on by a continued debauch. It is associated with prominent symptoms, flushed face, contracted pupil, and great nervous excitement. Delirium Tremens is developed in men who have been in a habit of drinking and who try to break it off. It is also induced in persons who have been moderate drinkers but who have received some severe shock, as fracture of the leg.

The treatment of this class of patients would not be so diverse if these differences in their character were more carefully noted. Persons suffering from Mania a potu are extremely excited and imagine some person or thing pursuing them, and under this hallucination will rush violently from the room or dash themselves headlong from the window unless carefully guarded. Not long since a man in this city imagining himself pursued by some men wishing to take his life, came into a saloon and asked protection. In a few moments he started up again, and passing out into the street stabbed a man whom he had never known or spoken to, to the heart.

Formerly persons suffering in this manner came to the Hospitals and were given opium and a small amount of alcoholic stimulant. This plan of treatment is mistaken. They should be placed in a moderately lighted and pleasant room where quiet can be insured, and given good light food. If this does not have the desired effect give as a purgative ten or twelve grains of calomel and a saline cathartic.

Sailors have a method of treating comrades suffering in this way by walking the subject between two of their number until he sleeps from physical exhaustion, but a mistake is some times made and the weak and tremulous sufferer from Delirium Tremens is made to walk in this way much to his detriment.

If purgatives do not have the desired effect give an anodyne, give a good dose of opium, or two or three grains of opium with a glass of whisky. Bromide of Pottassium seems to have, in some cases, a very happy effect; when they have taken twenty or thirty grains, you will often find the delirium has passed off and they awake with a good appetite.

In that form where exhaustion is the principal symptom we often find it beneficial to give a little stimulous with, perhaps, a little bitter infusion as Gentian, Columbo or Quassia, then give an anodyne.

The new anæsthetic, Hydrate of Chloral, is almost a specific in these cases in doses of fifteen or twenty grains; the patient wakes up with no nausea or headache, and perhaps with appetite. But you will often be disappointed to find that your patient will, during the day, have a return of his delusion, and you will, in some cases, be obliged to repeat your dose once in two or three hours. It is better and safer to give small repeated, rather than large single or large repeated doses. Deaths have often occurred from large doses of Hydrate of Chloral. It is a drug which is to be used with extreme caution.

